# Identification of mitochondria-related action targets of quercetin in melanoma cells

**DOI:** 10.1080/23802359.2023.2268775

**Published:** 2023-10-18

**Authors:** Qing Xi, Li Li, Yongjie Yang, Liubing Li, Rongxin Zhang

**Affiliations:** a Department of Gastroenterology, The First Affiliated Hospital of Guangdong Pharmaceutical University, Guangzhou, China; b School of Biomedical Sciences and Engineering, South China University of Technology, Guangzhou, China; c Department of Biotechnology, School of Life Sciences and Biopharmaceutics, Laboratory of Immunology and Inflammation, Guangdong Pharmaceutical University, Guangzhou, China; d Department of Laboratory Medicine, The First Affiliated Hospital, Sun Yat-sen University, Guangzhou, China

**Keywords:** Melanoma, mitochondrial dysfunction, quercetin, RNA-seq

## Abstract

Melanoma is a complex and genetically heterogeneous malignant tumor with high rates of mortality. Although current therapies provide a short-term clinical benefit, they are unable to cure the majority of patients with metastatic melanoma. Therefore, the investigation of pathological mechanisms and the development of new therapy strategies for melanoma are of great significance. Quercetin can effectively inhibit tumor growth in various tumors. However, the exact action mechanisms of quercetin against melanoma have not been comprehensively clarified, which limits its application. Accumulating evidence has suggested that the dysfunction of mitochondria is closely linked to carcinogenesis, and a better understanding of the regulation of mitochondria-related genes will shed light on providing new therapies for melanoma. In this study, we performed RNA-seq from melanoma B16-F1 cells treated with quercetin versus controls and screened for differentially expressed genes (DEGs). GO and KEGG enrichment analyses were performed, and a protein–protein interaction (PPI) network was constructed. Combining the results of RNA-seq, molecular docking, and bioinformatics analysis, we found six mitochondria-related genes, BTG2, CP, LRIG1, CYP1A1, GBP2, and MBNL1, which might be targets of quercetin in melanoma and provide an available targeting therapy strategy for melanoma.

## Introduction

Melanoma is the most aggressive skin cancer, with high rates of mortality (Saginala et al. 2021). Although current therapies provide a short-term clinical benefit, they are unable to cure the majority of patients with metastatic melanoma. Therefore, the development of new therapy strategies for melanoma is of great significance. Quercetin is a 5-hydroxyflavonol that belongs to the natural flavonoids (Yang et al. 2020), which play roles in antioxidant, anti-inflammatory, and immune regulation (Borghi et al. 2016), and can also inhibit tumorigenesis and progression (Reyes-Farias and Carrasco-Pozo 2019). However, the exact mechanisms of quercetin against melanoma have not been comprehensively clarified, which limits its application.

Mitochondria are ubiquitous organelles in eukaryotic cells, which play central roles in cell apoptosis (Wang 2001) and proliferation (Rustin 2002), and also act as major factors in regulating calcium signaling (Babcock and Hille 1998). Alterations in mitochondrial mechanisms, including the number, shape, and function of mitochondria, have been reported in various cancers and linked to carcinogenesis. The bioenergy conversion from mitochondrial oxidative phosphorylation to glycolysis has been considered to be a marker of tumor development or a bioenergy characteristic of cancer (Cuezva et al. 2002). Mitochondrial respiration defects in cancer cells lead to elevated levels of NADH, which could inactivate PTEN, enhance glycolysis, and increase cell survival (Pelicano et al. 2006). In addition, the mutations of mtDNA in cancers have critical impacts on tumorigenesis and metastasis (Ju et al. 2014). In this study, we found six mitochondria-related genes, BTG2, CP, LRIG1, CYP1A1, GBP2, and MBNL1, which might be targets of quercetin in melanoma and play crucial roles in melanoma.

## Materials and methods

Quercetin-treated B16-F1 cells and a control group were cleaved using TRIzol™ reagent, and then RNA-sequencing (RNA-seq) libraries were prepared and sequenced using Illumina NovaSeq 6000 (Guangzhou Genedenovo Biological Technology Co., Guangzhou, China). Differentially expressed genes (DEGs) were defined with a false discovery rate (FDR) of <0.05 and absolute fold change (FC) of ≥2. Heatmap, gene ontology (GO) and Kyoto encyclopedia of genes and genomes (KEGG) pathway analyses were performed, and a protein–protein interaction (PPI) network was constructed. Molecular docking was performed with PyMOL, AutodockTools, and AutoDock Vina (Trott and Olson 2010). The complete details of procedures are provided in the Supplementary Materials and Methods.

## Results and discussion

**Figure 1.**
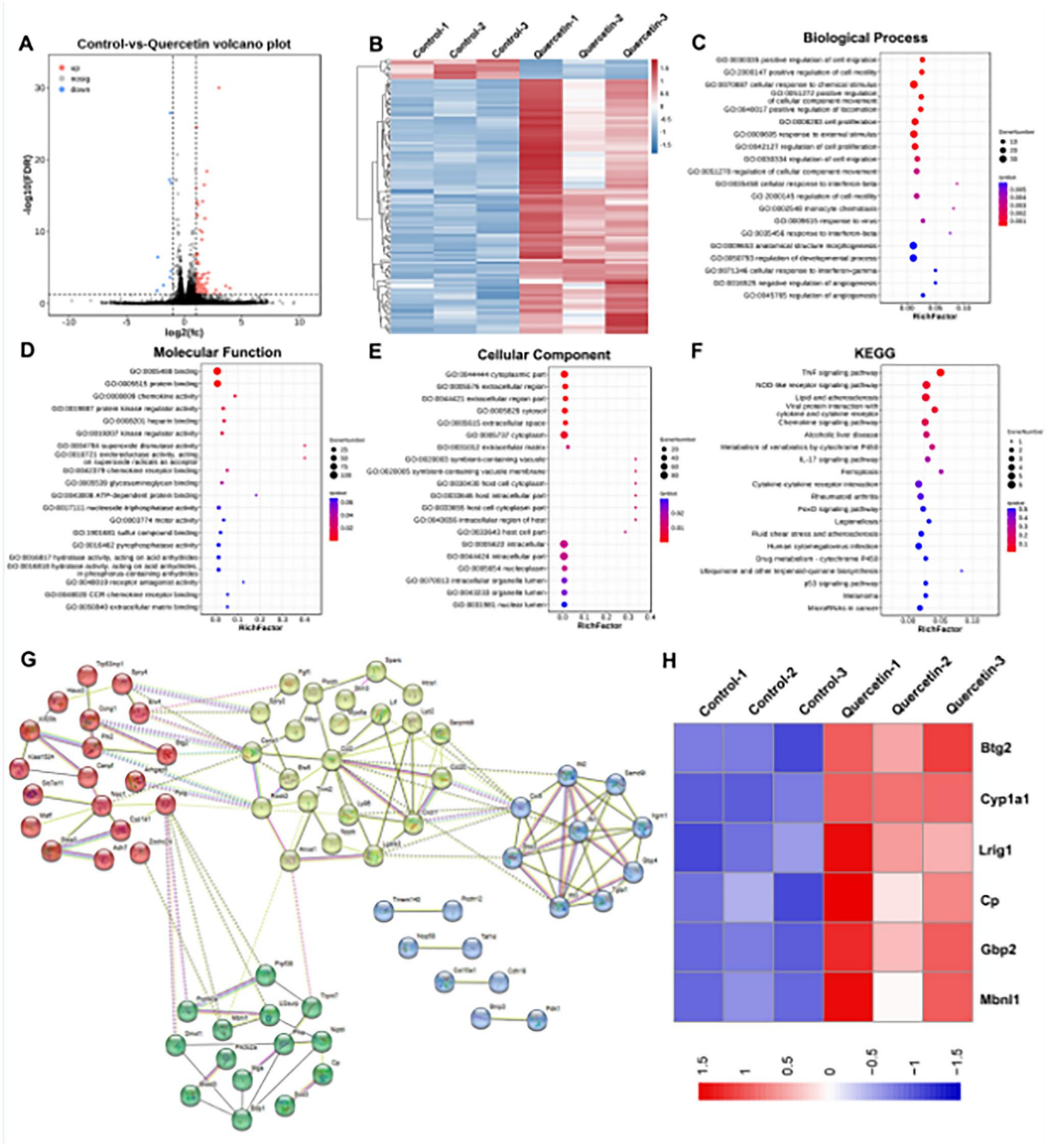
Identification of mitochondria-related action targets of quercetin. (A) Volcano plot of differentially expressed genes (DEGs) from melanoma B16-F1 cells treated with quercetin versus controls. DEGs are designated in red (up-regulation (up)) and blue (down-regulation (down)), having an FDR of less than 0.05. (B) Heat map of 110 DEGs. (C–F) The GO analysis (biological process, molecular function, and cellar component) and KEGG enrichment of DEGs. According to the *p* value, the top 20 pathways were selected to draw bubble charts. (G) The PPI network constructed for DEGs. (H) Heat map of mitochondria-related action targets of quercetin from RNA-seq.

**Figure 2.**
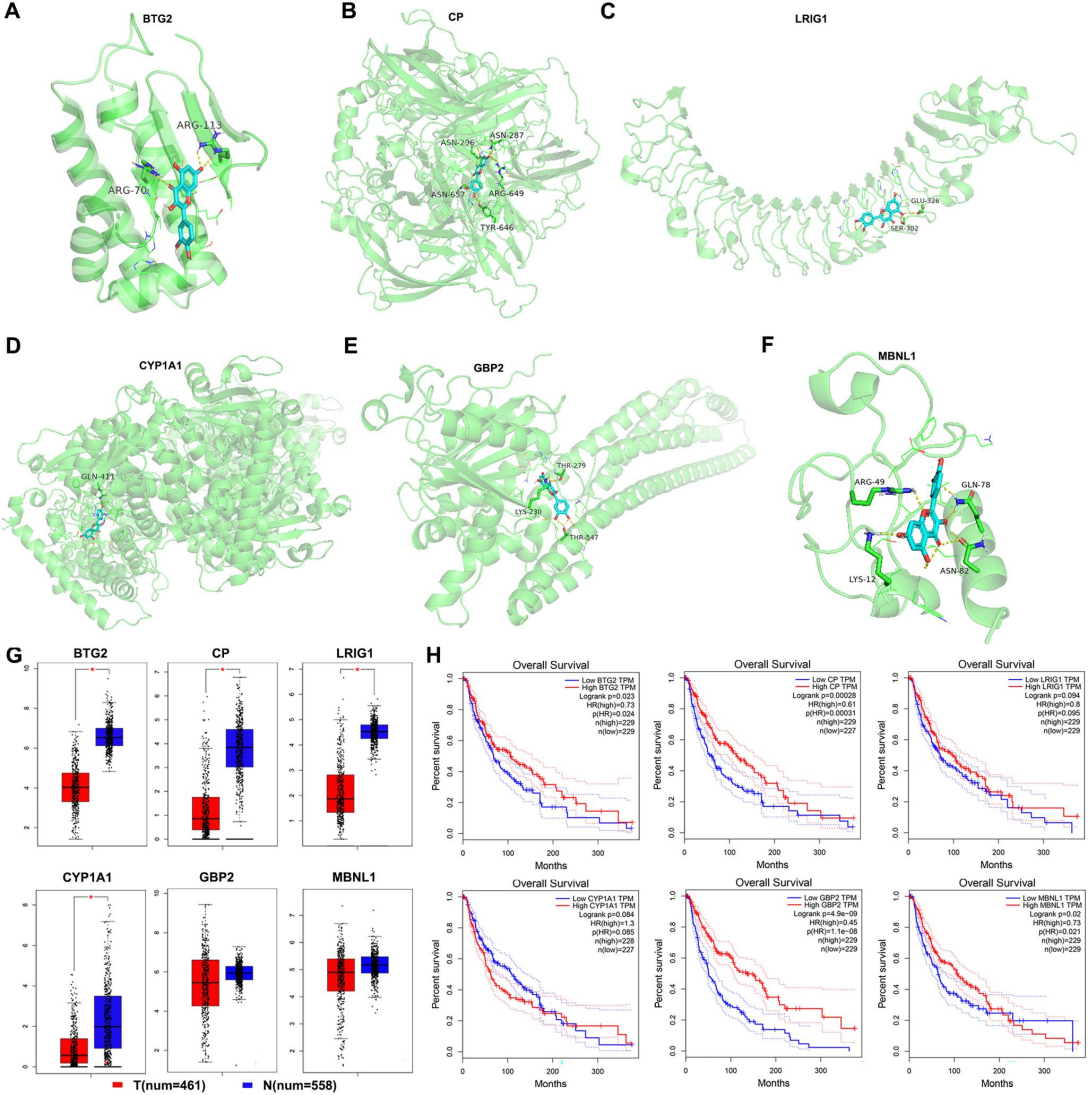
Assessment of mitochondria-related action targets of quercetin in melanoma. (A–F) Molecular docking of quercetin with BTG2, CP, LRIG1, CYP1A1, GBP2, and MBNL1, separately. (G) The expression of mitochondria-related action targets of quercetin. (H) Survival analysis of mitochondria-related action targets of quercetin.

In the present study, 110 DEGs (102 upregulated and eight downregulated) from quercetin-treated B16-F1 cells and the control group were identified. The volcano plot and heat map of all DEGs are shown in Figure 1(A,B). Subsequently, we performed GO analysis of the DEGs regarding three aspects, including biological process, molecular function, and cellar components (Figure 1(C–E)), and the pathways were mainly concentrated in cellular response to chemical stimuli, cell proliferation, cell migration, response to external stimuli, and other activities. Most DEGs were correlated with binding and protein binding, and they were mainly involved in the cytoplasm and cytoplasmic parts. We also performed KEGG enrichment of DEGs (Figure 1(F)), and multiple signaling pathways, including TNF-α, the NOD-like receptor, chemical signaling pathway, metabolism of xenobiotics by cytochrome P450, and other signaling pathways, were closely related to the action of quercetin. Then, a PPI network with 109 nodes and 132 edges was constructed according to the STRING database, with an interaction score of >0.4 (Figure 1(G)). We further analyzed the expression of mitochondrial-related DEGs, and the heat map showed that Btg2, Cp, Lrig1, Cyp1a1, Gbp2, and Mbnl1 were upregulated by quercetin (Figure 1(H)). Furthermore, we performed molecular docking to investigate whether or not quercetin can bind to the proposed target proteins (Figure 2(A–F)). The results indicate that quercetin can bind to BTG2, CP, LRIG1, CYP1A1, GBP2, and MBNL1, with binding affinities of −6.6, −8.7, −7.1, −8.9, −7.7, and −6.57 kcal/mol, respectively. Then, we analyzed the expression of these mitochondria-related genes and their relationship with overall survival in melanoma using the online Gene Expression Profiling Interactive Analysis (GEPIA) database (Tang et al. 2017). The expressions of the mitochondrial-related genes BTG2, CP, LRIG1, and CYP1A1 were lower in melanoma than in normal tissues, while the expression levels of GBP2 and MBNL1 did not show a statistically significant difference between melanoma and normal tissues (Figure 2(G)). Moreover, the higher the expression of BTG2, CP, GBP2, and MBNL1, the better the prognosis; while the prognosis of LRIG1 and CYP1A1 did not have statistical significance (Figure 2(H)).

Previously, BTG2 down-expression was found to promote cellular lipid accumulation, proliferation, and invasion, accompanied by aggravated mitochondrial dysfunction, including increased fading and ROS products of mitochondria (Su et al. 2022). Additionally, BTG2 negatively regulated cancer cell migration through downregulation of ROS generation in mitochondria (Lim et al. 2012). In this study, Btg2 increased after quercetin treatment, which might ameliorate cellular lipid accumulation, slow cell proliferation and invasion, and ameliorate mitochondrial dysfunction, effectively reversing lipotropic and oncogenic effects. BTG2 has a lower expression in melanoma, and the lower the expression of BTG2, the worse the prognosis. Therefore, we predicted that BTG2 might be a tumor marker closely related to melanoma. Another critical gene, ceruloplasmin (CP), is a major cuproenzyme considered the major systemic copper carrier, which is associated with mitochondrial dysfunction. Previous studies have shown that reduced expression of CP predicts poor prognosis in adrenocortical carcinoma (Zhu et al. 2019), and it could inhibit the proliferation, migration, and invasion of nasopharyngeal carcinoma cells (Yang et al. 2022). Our findings suggested that CP has a lower expression in melanoma, and reduced CP expression might predict a poor prognosis in patients suffering from melanoma. The expression of Cp increased after quercetin treatment, which may correlate with mitochondrial dysfunction, though the exact function and mechanism need to be elucidated. The leucine-rich repeats and immunoglobulin-like domains 1 (LRIG1) is the natural ligand of EGFR, resulting in downregulation of EGFR expression. LRIG1 inhibited the EGFR signaling pathway and activated the mitochondrial pathway of apoptosis (Yan et al. 2015). In this study, Lrig1 increased after quercetin treatment in B16-F1 cells, and had a lower expression in melanoma, which might regulate tumor cell apoptosis through impacting the mitochondrial pathway. CYP1A1, as a bioactivating enzyme responsible for generating a metabolite capable of damaging DNA via adduct formation, is closely related to mitochondrial homeostasis, energy metabolism, and carcinogen metabolizing pathway. Previous studies reported that macrophages attenuate the transcription of CYP1A1 in tumor cells and enhance their proliferation (Winslow et al. 2019), and knockdown of CYP1A1 attenuated the extractable organic matter (EOM)-induced mitochondrial dysfunction, including increased mitochondrial ROS (mtROS) levels, mitochondrial permeability transition pore (mPTP) opening, and mitochondrial membrane potential (MMP) collapse, and it reduced mitochondrial ATP levels (Chen et al. 2023). In this study, Cyp1a1 increased after being treated with quercetin, and had a lower expression in melanoma, which indicated that quercetin might regulate tumor cell biological function through CYP1A1 via the mitochondrial pathway. Previously, guanylate-binding protein 2 (GBP2) was found to attenuate Drp1-mediated mitochondrial fission to suppress the invasion of cancer cells (Zhang et al. 2017). In this study, Gbp2 increased after quercetin treatment in B16-F1 cells. The higher expression of GBP2 indicated better progression, which indicated that GBP2 may play critical roles in melanoma through regulating mitochondrial fission to suppress invasion. Moreover, muscle blind-like 1 (MBNL1) is a class of conserved splicing factors that play important roles in the development of multiple tissues and cancers (Lin et al. 2006; Voss et al. 2020). MBNL1 is closely related to mitochondrial dysfunction and apoptosis (Yokoyama et al. 2020). In this study, Mbnl1 increased after being treated with quercetin. The higher expression of MBNL1 indicated better progression, which indicated that MBNL1 may play critical roles in melanoma through regulating mitochondrial function and apoptosis.

Collectively, we demonstrated that the six mitochondria-related genes, BTG2, CP, LRIG1, CYP1A1, GBP2, and MBNL1, might be targets of quercetin in melanoma, which provides novel insights into the drug discovery and treatment of melanoma.

## Supplementary Material

Supplemental Material

Supplemental Material

## Data Availability

The RNA-seq data that support the findings of this study are openly available in NCBI at https://www.ncbi.nlm.nih.gov/sra/PRJNA952519. The associated BioProject accession number is PRJNA952519; the Bio-Sample numbers are SAMN34074959, SAMN34074960, SAMN34074961, SAMN34074962, SAMN34074963, and SAMN34074964; the SRA accession numbers are SRR24071997, SRR24071996, SRR24071995, SRR24071994, SRR24071993, and SRR24071992.
