# Supplementary material for: Identification of mitochondria-related action targets of quercetin in melanoma cells
Source: Mitochondrial DNA B Resour. 2023 Oct 18;8(10):1114–8. doi: 10.1080/23802359.2023.2268775 (PMC10586065; doi:10.1080/23802359.2023.2268775)
Supplement: Supplemental Material [file TMDN_A_2268775_SM7123.docx]

**Identification of mitochondria-related action targets of quercetin**

**in melanoma cells**

**Supplementary materials and methods**

### Cell culture and drug treatment

The mouse melanoma B16-F1 cells were original obtained from ATCC (American Type Culture Collection, USA).The cells were cultured in Dulbecco's Modified Eagle Medium (Gibco, USA) containing, 10% fetal bovine serum (FBS; Procell, China), 100 U/mL penicillin and 100 µg/mL streptomycin, and incubated at 37℃ and under 5% CO_2_ in culture dishes in a humidified incubator (Thermo Fisher Scientific, USA). The cells were collected for analysis upon reaching 80% confluence. Quercetin was purchased from MedChemExpress (MCE, USA, HY-18085) with a purity of above 98%, and dissolved in [dimethyl sulfoxide](https://www.sciencedirect.com/topics/medicine-and-dentistry/dimethyl-sulfoxide) (DMSO) to treat B16-F1 cells.

**RNA-sequencing**

Quercetin treated B16-F1 cells and control group were cleaved by TRIzol™ reagent, the mRNA was enriched by Oligo(dT) beads after total RNA was extracted, and fragmented into short fragments, then reversly transcribed into cDNA. The purified double-stranded cDNA fragments were end repaired, A base added, and ligated to Illumina sequencing adapters, then RNA-sequencing (RNA-seq) libraries were prepared and sequenced using Illumina NovaSeq 6000 in Guangzhou Genedenovo Biological Technology Co. Differentially expressed genes (DEGs) were defined with false discovery rate (FDR) <0.05 and absolute fold change(FC)≥2.

### Pathway and protein-protein interaction (PPI) network analysis

Gene Ontology (GO) and Kyoto Encyclopedia of Genes and Genomes (KEGG) pathway enrichment analyses were conducted to predict gene functions at three levels: [Molecular function](https://www.sciencedirect.com/topics/computer-science/molecular-function) (MF), [Biological process](https://www.sciencedirect.com/topics/medicine-and-dentistry/biological-phenomena-and-functions-concerning-the-entire-organism) (BP), Cellular component (CC), as well as KEGG pathways. The top 20 GO enrichment and top 20 KEGG pathways were chosen to draw bubble charts. An online STRING database (https://cn.string-db.org/) was used to create the Protein-Protein Interaction (PPI) network of target genes, the network of PPI was then imported into Cytoscape v.3.9.0 for the identification of hub genes.

### Molecular docking

### The 3D structure of the mitochondria-related protein was obtained from the RCSB PDB database (<https://www.rcsb.org/>). For the molecule ligands, the 2D structure of quercetin was retrieved from the [PubChem](https://www.sciencedirect.com/topics/medicine-and-dentistry/pubchem) database (<https://pubchem.ncbi.nlm.nih.gov/>). AutodockTools and AutoDock Vina were used to add polar hydrogen and predict the binding between the mitochondria-related proteins and quercetin. The results of the binding poses of quercetin were visualized using PyMOL.

### Gene expression and survival analysis

### The survival analysis of mitochondria-related genes was carried out utilizing GEPIA2 (<http://gepia2.cancer-pku.cn/>). The clinical importance of a particular gene is also assessed using survival analysis based on gene expression levels. Kaplan-Meier survival curves were constructed using the database for skin cutaneous melanoma (SKCM) to examine the relationship between genes and overall survival of SKCM [patients](https://www.sciencedirect.com/topics/medicine-and-dentistry/patient). P value <0.05 were assumed significant statistically.
