# Supplementary material for: Identification of mitochondria-related action targets of quercetin in melanoma cells
Source: Mitochondrial DNA B Resour. 2023 Oct 18;8(10):1114–8. doi: 10.1080/23802359.2023.2268775 (PMC10586065; doi:10.1080/23802359.2023.2268775)
Supplement: Supplemental Material [file TMDN_A_2268775_SM7110.pdf]

We certify that the following article

Identification of mitochondria-related action targets of quercetin in melanoma cells

Rongxin Zhang

has undergone English language editing by MDPI. The text has been checked for correct use of grammar and common technical terms, and edited to a level suitable for reporting research in a scholarly journal.
MDPI uses experienced, native English speaking editors. Full details of the editing service can be found at
► https://www.mdpi.com/authors/english.

MDPI Multidisciplinary Digital Publishing Institute

Basel, Switzerland
September 2023

AUTHORservices

english-71258
